# Whole Genome Sequencing Solves an Atypical Form of Bardet–Biedl Syndrome: Identification of Novel Pathogenic Variants of *BBS9*

**DOI:** 10.3390/ijms25158313

**Published:** 2024-07-30

**Authors:** Emilia Stellacci, Marcello Niceta, Alessandro Bruselles, Emilio Straface, Massimo Tatti, Mattia Carvetta, Cecilia Mancini, Serena Cecchetti, Mariacristina Parravano, Lucilla Barbano, Monica Varano, Marco Tartaglia, Lucia Ziccardi, Viviana Cordeddu

**Affiliations:** 1 Dipartimento di Oncologia e Medicina Molecolare, Istituto Superiore di Sanità, 00161 Rome, Italy; emilia.stellacci@iss.it (E.S.); alessandro.bruselles@iss.it (A.B.); strafaceemilio8@gmail.com (E.S.);; 2 Genetica Molecolare e Genomica Funzionale, Ospedale Pediatrico Bambino Gesù, IRCCS, 00146 Rome, Italy; marcello.niceta@opbg.net (M.N.); mattia.carvetta@opbg.net (M.C.); cecilia.mancini@opbg.net (C.M.); marco.tartaglia@opbg.net (M.T.); 3 Confocal Microscopy Unit—Core Facilities, Istituto Superiore di Sanità, 00161 Rome, Italy; serena.cecchetti@iss.it; 4 Fondazione Bietti, IRCCS, 00198 Rome, Italy; mariacristina.parravano@fondazionebietti.it (M.P.); lucilla.barbano@fondazionebietti.it (L.B.); monica.varano@fondazionebietti.it (M.V.)

**Keywords:** whole genome sequencing, *BBS9*, structural variant, Bardet–Biedl syndrome, ciliopathy

## Abstract

Bardet–Biedl syndrome (BBS) is a rare recessive multisystem disorder characterized by retinitis pigmentosa, obesity, postaxial polydactyly, cognitive deficits, and genitourinary defects. BBS is clinically variable and genetically heterogeneous, with 26 genes identified to contribute to the disorder when mutated, the majority encoding proteins playing role in primary cilium biogenesis, intraflagellar transport, and ciliary trafficking. Here, we report on an 18-year-old boy with features including severe photophobia and central vision loss since childhood, hexadactyly of the right foot and a supernumerary nipple, which were suggestive of BBS. Genetic analyses using targeted resequencing and exome sequencing failed to provide a conclusive genetic diagnosis. Whole-genome sequencing (WGS) allowed us to identify compound heterozygosity for a missense variant and a large intragenic deletion encompassing exon 12 in *BBS9* as underlying the condition. We assessed the functional impact of the identified variants and demonstrated that they impair BBS9 function, with significant consequences for primary cilium formation and morphology. Overall, this study further highlights the usefulness of WGS in the diagnostic workflow of rare diseases to reach a definitive diagnosis. This report also remarks on a requirement for functional validation analyses to more effectively classify variants that are identified in the frame of the diagnostic workflow.

## 1. Introduction

Bardet–Biedl syndrome (BBS, MIM #209900) is a rare recessive multisystem disorder characterized by retinitis pigmentosa, obesity, postaxial polydactyly, cognitive deficits, and genitourinary defects [1,2,3]. Other features that commonly occur include developmental delay, speech disorder or delay, ataxia, poor coordination, strabismus, cataracts, dental crowding, hypodontia, small dental roots or high arched palate, brachydactyly/syndactyly, polyuria/polydipsia (nephrogenic diabetes insipidus), diabetes mellitus, left ventricular hypertrophy or congenital heart disease, and/or liver fibrosis [2].

BBS is characterized by clinical variability, which is mirrored by the considerable genetic heterogeneity characterizing the disorder. To date, 26 genes have been identified to be implicated in BBS, which encode proteins involved in the formation, size, and function of the primary cilium, as well as in intraflagellar transport and ciliary trafficking. Among them, genes designated *BBS1* to *BBS21* account for approximately 80% of BBS cases [2,3,4].

BBS1, BBS2, BBS4, BBS5, BBS7, BBS8, BBS9, and BBS18 form a hetero-octameric complex called the BBSome, which plays a central role in the formation and function of primary cilia [5]. The other members of the BBS protein family, in turn, mediate BBSome assembly (i.e., BBS6, BBS10, and BBS12), ciliary transport (i.e., MKS1/BBS13, IFT27/BBS19 and LZTFL1/BBS17), ciliogenesis (i.e., WDPCP/BBS15, SDCCAG8/BBS16, and SCLT1), intraflagellar transport (i.e., IFT27 and IFT74/BBS20), and ciliary entry (CEP290/BBS14) and dynamics (SCAPER) [3]. During BBSome formation, BBS2 and BBS7 form a tight dimer that associates with BBS9 to build the BBS2-BBS7-BBS9 core [6,7]. BBS1, BBS5, and BBS8 are then recruited to the BBSome via direct association with BBS9, and, finally, BBS4 binds to the BBSome. Therefore, BBS9 can be considered as a key element of the BBSome acting as an aggregator for recruiting the other members of the complex, organizing a core sub-complex together with BBS1, BBS2, and BBS7. BBS8 and BBS18 function as linkers between BBS4 and BBS9 at the periphery, while BBS5 localizes to the periphery of the core complex to bind the membrane [6,7]. Since photoreceptors are specific neurons located on the outer portion of the retina and are equipped with specialized cilia, any alteration of the BBSome may be accountable for an altered structural organization of photoreceptor outer segments, abnormal signaling, and ciliary defects, leading to the broad subset of disorders known as ciliopathies [3]. These findings are consistent with the fundamental role of the BBSome and its localization in the basal body of the primary *cilium* [3,8].

This work describes a young adult who had a history of severe photophobia and central vision loss since childhood and shows hexadactyly of the right foot and a supernumerary nipple at birth, which were suggestive of BBS. Molecular analyses by targeted resequencing and whole-exome sequencing (WES) failed to reach a definitive molecular diagnosis. Whole-genome sequencing (WGS) revealed compound heterozygosity for two variants, a missense one and a large intra-genic deletion, in the *BBS9* gene as the putative cause of the condition. Structural and functional analyses were used to validate the functional relevance of the two variants.

## 2. Results

### 2.1. Clinical Report

The proband was an 18-year-old subject who reported severe photophobia and central visual loss since childhood. He was the first child of three born to nonconsanguineous parents of European descent. The father’s ophthalmic examination showed signs of central serous chorioretinopathy without electroretinographic or visual field defects specific for retinal dystrophies. The younger brother had polycystic kidney disease and no evidence of retinal damage. No other family members (including the mother and younger sister) were affected by retinal dystrophies or other congenital defects.

The subject was born by vaginal delivery in the 39th week of an uneventful pregnancy. Birth weight was 2750 g (−1.8 Standard Deviation, SD), length was 50 cm (−0.44 SD), and head circumference (occipitofrontal circumference, OFC) was 34 cm (−0.7 SD). Developmental delay was not recorded in his early childhood. The subject was first evaluated at 8 years of age because of severe photophobia and central vision loss in both eyes (oculus uterque, OU). Supernumerary nipple and postaxial polydactyly (hexadactyly) at the right foot were also noticed. The rudimentary digit was surgically removed. His visual acuity was 20/80 Snellen in the right (RE) and the left (LE) eye, with myopic and astigmatic refractive errors (RE: −1.50 diopter sphere and −1 diopter cylinder axis 90°; LE: −0.75 diopter sphere and −1.00 diopter cylinder axis 90°). Abnormal color vision was assessed by Ishihara charts in the OU (RE: 5/22 charts; LE: 2/22 charts). The slit lamp biomicroscopy of the anterior segment was normal. Fundus examination in OU showed retinal pigment epithelium (RPE) dystrophy in the macular area with no intraretinal pigment dispersion in the peripheral retina. Goldmann’s visual field showed slight temporal constriction and a small central scotoma in OU. Light- and dark-adapted full-field flash electroretinograms showed reduced retinal function, and light- and dark-adapted sensitivity curves showed abnormal cone and rod thresholds. Multifocal electroretinogram (mfERG) ring analysis documented reduced response amplitude densities in all areas examined from the foveal center to 20° eccentricity in the OU. The spectral-domain optical coherence tomography (sd-OCT) showed a rarefaction of the subfoveal ellipsoid zone suggestive of cone dystrophy. Subsequently, the ophthalmic condition appeared stable for about 7 years.

**Figure 1:**
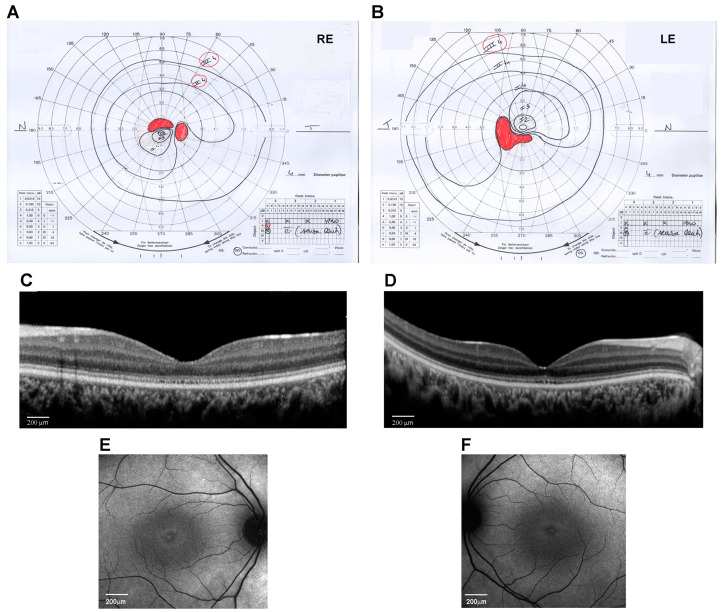
Ocular phenotype of the proband eyes (Right Eye, RE, and Left Eye, LE). (**A**,**B**) Kinetic visual field test by Goldmann perimeter (Haag–Streit, Bern, Switzerland). Black lines are the isopters that delineate the analyzed visual field area. Red areas indicate the scotoma and blind spot areas. (**C**,**D**) Spectral-domain optical coherence tomography (OCT) line horizontal scan by Spectralis (Heidelberg Engineering, Heidelberg, Germany) (scale bar 200 μm). (**E**,**F**) 30° Fundus autofluorescence imaging of the central area by Spectralis (Heidelberg Engineering, Heidelberg, Germany) (scale bar 200 μm).

At the age of 14 years, his weight was 50 kg (0.09 SD), height was 148 cm (−1.73 SD), and OFC was 56 cm (−0.51 SD). The subject did not show signs of obesity. Physical growth and sexual maturation were normal. Generalized hypotrichosis was noticed. No cognitive deficits, behavioral abnormalities, or facial dysmorphism were observed. Ophthalmological examination revealed a progressive decrease in visual acuity to 20/200 in both RE and LE with myopic and astigmatic refractive errors (RE: −1.00 diopter sphere and −0.75 diopter cylinder axis 30°; LE: −0.75 diopter sphere and −1.00 diopter cylinder axis 130°) and reduced color vision (Ishihara charts: 1/22 in OU). Adaptive optics testing was assessed, which allowed for the quantification of foveal cone rarefaction in OU. Goldmann’s visual field (Figure 1A,B) showed a larger scotomatous area in the superior (RE) and inferior (LE) fields, in addition to the central scotoma in OU. Dark-adapted and light-adapted full-field electroretinograms showed reduced a- and b-wave amplitudes and delayed implicit times. Light- and dark-adaptation sensitivity curves confirmed abnormal thresholds for both cones and rods. Sd-OCT scans showed a thinning of all outer and inner retinal layers (Figure 1C,D) and the disruption of the subfoveal ellipsoid zone, and fundus autofluorescence (AF) imaging of the central 30° area showed a perifoveal hyperautofluorescent ring in OU (Figure 1E,F). MfERG analysis showed a further reduction in response amplitude densities in all rings of OU. Subsequent adaptive optics testing allowed for the further quantification of foveal cone rarefaction in OU. Goldmann’s visual field examination showed progressive concentric peripheral constriction of the peripheral isopters and an enlargement of the central scotoma, which is typical of progressive cone–rod dystrophy.

At last evaluation, at 18 years, his weight was 57 kg (0.09 SD), height was 167.8 cm (−1.73 SD), and OFC was 55 cm (−0.51 SD). The remaining physical assessment was unremarkable. Abdominal ultrasonography and audiological evaluations were normal. For the first time, the blood count, TSH, free triiodothyronine, free thyroxine, insulin, IGF1, and GH were performed and were all normal.

### 2.2. Genomic Analyses

Since diagnostic routine second-generation sequencing performed on the trio considering a gene panel opportunely designed for retinal disorders was not conclusive, we first performed WES on the trio. Due to the variable coverage of individual coding exons of the *BBS9* gene (average coverage of 81, with poor coverage for coding exons 3, 6, 10, 12, 18, and 21 [coverage lower than 40×]), we were only able to identify a heterozygous missense variant in the *BBS9* gene (c.545G>C, p.Gly182Ala, NM_198428.3, ENST00000242067.11) in the proband and his mother, which had not previously been reported in ClinVar (gnomAD v4.0, MaxAF = 0.000004). Although this variant was classified as of uncertain significance (VUS), it was considered potentially clinically relevant because it involves the β-propeller domain, which specifically mediates protein–protein interactions, and because biallelic loss-of-function (LoF) variants in *BBS9* cause BBS. Indeed, a more accurate clinical assessment of the proband documented the occurrence of some distinctive features of the syndrome, including hexadactyly and a supernumerary nipple, which had not originally been recognized. As no other functionally relevant variants associated with known BBS disorder features were identified based on the proband’s clinical presentation and inheritance model, and considering that the form of BBS associated with this gene is inherited in an autosomal recessive pattern [1,2,4], WGS was performed on the proband and his parents to investigate the occurrence of a second hit involving the other allele. Besides the previously identified maternally inherited missense variant, WGS analysis led us to identify a private intronic deletion of ~1.2 Kb within the *BBS9* gene (chr7:33348318-33349583del). The detailed variant calling and WGS metrics are reported in Supplementary Table S1.

### 2.3. Functional Validation Analyses

**Figure 2:**
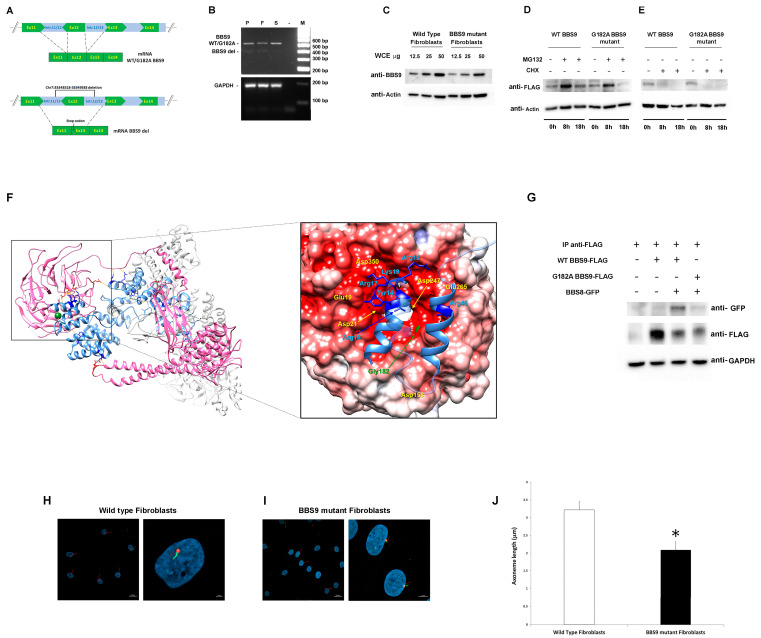
Characterization of the pathogenic *BBS9* variants. (**A**) Schematic representation of aberrant transcript processing caused by the identified intronic deletion encompassing Exon 12 in *BBS9*. (**B**) Agarose gel analysis of transcript processing in primary skin fibroblasts from the proband (P) carrying the biallelic variants and from peripheral blood mononuclear cell (PBMC) of the father (F) and the sister (S). Total RNA was extracted, reverse-transcribed, and analyzed by PCR to resolve transcript processing. Two cDNA products of 583 and 480 base pairs were identified in P and F, respectively, and one of 583 bp in S and sequenced. GAPDH was used as control. (**C**) Western blot analysis of the BBS9 protein in patient (carrying both identified variants) and control fibroblasts. Twelve and a half, twenty-five, and fifty micrograms of whole cell extract (WCE) were analyzed and probed with anti-BBS9 and anti-Actin (as internal control) antibodies. (**D**) HEK293T cells, transiently transfected with WT BBS9 or G182A BBS9 mutant FLAG-tagged, basally and following 8 h and 18 h treatment with 10 μM MG132, were probed with anti-FLAG and anti-Actin (as loading control) antibodies. (**E**) HEK293T cells, transiently transfected with WT BBS9 or G182A BBS9 mutant FLAG-tagged, basally and following 8 h and 18 h treatment with 50 µg/mL cycloheximide (CHX) were probed with anti-FLAG and anti-Actin (as loading control) antibodies. (**F**) Structural model of BBS Subunits 1, 4, 8, 9, and 18 of the human BBSome complex (PBD: 6XT9). BBS9 is shown in hot pink and BBS8/TTC8 in cornflower blue. Side chains of BBS9 and BBS8/TTC8 residues involved in the intermolecular H-bond network (orange segments) are shown in red and blue, respectively. Gly182 is shown in forest green as a mass-weighted spherical centroid. The interacting surfaces of BBS9 (region of the β-propeller domain in which Gly182 is located) and BBS8/TTC8 are reported on the right panel. The structure has been rotated to show the surface of the β-propeller domain colored by its electrostatic potential (gradient ranging from red [negative charge] to blue [positive charge]). In the enlarged panel, side chains of the BBS8/TTC8 residues contributing to the intermolecular H bonding network are shown in blue. Residues directly involved in these interactions are indicated, whereas Residues 21 to 37 and 50 to 60 are rendered semitransparent for clarity. (**G**) Co-Immunoprecipitation analyses. As indicated, HEK293T cells were transfected with WT BBS9 or G182A BBS9 mutant FLAG-tagged and BBS8 GFP-tagged constructs (+ and − symbols show the presence or absence of the corresponding construct), and 500 micrograms of WCE were immunoprecipitated with anti-FLAG antibodies (see IP anti-FLAG row) analyzed by Western blot and then probed with anti-GFP (indicating the presence/absence of BBS9/BBS8 complex), anti-FLAG (indicating successful immunoprecipitation of BBS9), and anti-GAPDH (as loading control) antibodies. All blots are representative of three experiments performed. (**H**,**I**). Cilium defects in primary cells with BBS9 LoF. Fibroblasts from a healthy donor (**H**) and from the proband (**I**) were analyzed by confocal microscopy for the presence of primary cilia. Cells were starved for 30 h and fixed with 4% paraformaldehyde (PFA). Primary cilia were analyzed using antibodies against ARL13B (cilium axonemal, green) and pericentrin (basal body, red) to investigate morphogenesis. Nuclei are DAPI stained (blue). Bars correspond to 20 µm for panel showing multiple cells ((**H**,**I**) left panel) and 2 μm for panel showing one or two representative cells ((**H**,**I**) right panel). (**J**) The bar graphs represent the mean axoneme length in normal fibroblasts ((**H**), 3.22 μm ± 0.37 μm SD) compared to mutant fibroblasts ((**I**), 2.09 μm ± 0.24 μm SD). Five hundred cells were counted for each condition, and axoneme length was measured. Imagines and bar graphs are representative of three experiments performed. * Student’s *t*-test, *p* < 0.01.

*BBS9* spans 23 exons and encodes a protein of 887 amino acid residues (UniProtID: Q3SYG4 PTHB1_HUMAN). The identified deletion included the entire exon 12, plus a large intronic portion (Figure 2A), and was predicted to cause a frameshift in the coding sequence, resulting in a shortened protein. To validate the intragenic deletion, a PCR-based assay was designed to amplify and sequence the *BBS9* cDNA region encompassing the deletion to characterize transcript processing. As an RNA source, primary fibroblasts were isolated from a skin biopsy obtained from the subject and cultured, while peripheral blood was used from relatives. Total RNA was retro-transcribed into cDNA, and the region between Exons 9 and 15 of *BBS9* was amplified and analyzed. In line with an expected product lacking Exon 12, the agarose-gel analysis revealed two bands (583 and 480 bp) that were sequenced (Figure 2B). The sequencing of the low molecular weight (MW) amplified products shared by the proband, and his father documented a deletion of 103 bp corresponding to the skipping of the entire Exon 12. The skipping caused a frameshift starting with Exon 13, which introduced a stop codon, resulting in a truncated protein lacking the γ-adaptin ear (GAE) domain. Due to the lower abundance compared to the wild-type (WT) allele, it is possible that the mutated mRNA is not stable and undergoes accelerated degradation. The sequencing of the high MW cDNA product confirmed the presence of the Gly182Ala missense change in the proband and his unaffected sister.

To validate the functional consequences of the p.Gly182Ala substitution, we first assessed its possible impact on protein levels and stability using primary fibroblasts of the proband and transiently transfected HEK293T cells. Western blot (WB) analysis showed lower BBS9 levels in patient-derived fibroblasts compared to control cells, probably due to reduced synthesis and/or accelerated degradation of the protein lacking the portion encoded by Exon 12 (Figure 2C). Transient transfection experiments in HEK293T cells showed comparable degradation and stability between the p.Gly182Ala BBS9 mutant and the WT protein. WB analysis using transfected HEK293T cells treated with MG132 (which blocks proteasome-mediated protein degradation) and with cycloheximide (CHX, which blocks protein synthesis) confirmed that the p.Gly182Ala substitution does not alter degradation (Figure 2D) and protein stability (Figure 2E). Since the Gly-to-Ala substitution at Codon 182 affects the β-propeller domain, which mediates the binding of BBS9 to BBS8 (also known as TTC8) in the BBSome. We hypothesized a possible impact of the amino acid substitution on the protein–protein binding properties of the mutant. Notwithstanding that this residue does not directly contribute to the intermolecular binding network stabilizing the BBS9-BBS8/TCC8 interaction, it is located close to the surface of the domain that binds to BBS8/TTC8 (Figure 2F). A number of residues participating in the intermolecular H-bonds are spatially close to Gly182 (Glu19, Asp21, Asp158, Asp247, Glu265, Asp350), suggesting the possibility that a local conformational rearrangement of the domain might perturb the stability of these interactions. To validate this hypothesis, co-immunoprecipitation (co-IP) experiments were performed in transfected HEK293T cells. As shown in Figure 2G, the results indicated a defective interaction between p.Gly182Ala BBS9 and BBS8/TTC8. Taken together, our results suggest that the missense variant does not significantly alter the expression, degradation, and stability of BBS9. However, it perturbs the proper binding of the protein to BBS8/TTC8, affecting the assembly of the BBsome.

Based on the documented role of BBS9 in ciliogenesis, as a core element of the BBSome, and the demonstrated LoF behavior of the two identified *BBS9* variants (p.Gly182Ala and chr7:33348318-33349583del), confocal microscopy analysis was performed in primary fibroblasts to investigate cilium formation and morphology using antibodies against ARL13B, which localizes to the primary *cilium*, and pericentrin, a component of the centrosome, which stains the basal body. Compared to control cells, virtually all fibroblasts carrying the biallelic variants in *BBS9* were characterized by a significantly shorter primary *cilium* (respectively, 3.22 μm ± 0.37 μm SD vs. 2.09 μm ± 0.24 μm SD, Figure 2H,I).

### 2.4. Clinical Comparison with Features of Bardet-Biedl Syndrome 9

The clinical features of the proband were compared with those of 13 previously reported patients with biallelic pathogenic variants, either truncating or missense changes, in the *BBS9* gene (Supplementary Table S2). Retinal dystrophy, bilateral polydactyly in hands and feet, obesity, ID/DD, and genitourinary malformations were almost invariably observed in these subjects, resuming the cardinal features of BBS disorder. However, we noted that the individuals with missense variants, all of which affect the β-propeller domain (e.g., p.Gly141Arg, p.Gly243Lys, and the present variant p.Gly182Ala), were characterized by a milder phenotype including retinopathy and polydactyly (unilateral or in both feet), with or without obesity or multiorgan anomalies [9].

## 3. Discussion

The application of genome scanning in clinical practice is widely recognized as a diagnostic milestone. The use of WES as a first-tier diagnostic tool has significantly improved the diagnostic yield for patients affected by rare diseases. Nevertheless, WES has been shown to be uninformative in a large fraction of cases due to both technical limitations and the nature of the genomic lesion(s) underlying the disease. Recently, the applications of WGS have been used in a constellation of research programs to approach the aforementioned challenges, supporting their efficacy in improving the diagnosis in rare diseases [10,11,12,13,14,15]. While WGS is not included in the routine diagnostic workflow, it allows for the identification of deep intronic variants affecting transcript processing, variants within regulatory, and 5′- and 3′-untranslated regions impacting RNA transcription, stability, and translation, as well as structural variants that are not systematically inspected by WES. The case presented in this study confirms the importance of using WGS in patients where targeted resequencing or WES fails to reach a molecular diagnosis.

When the subject was clinically reassessed, in addition to the severe photophobia, central vision loss since infancy and reported hexadactyly of the right foot, which had been corrected in infancy, as well as the occurrence of a supernumerary nipple, were disclosed. Overall, these features are suggestive of BBS. However, he did not show any of the other typical features characterizing the disorder, such as obesity, kidney problems or malformations, and intellectual disability. BBS is a ciliopathy, a class of genetic disorders characterized by ciliary dysfunction. They are caused by the defective function of several proteins involved in ciliary structure and/or function, as well as in pathways and complexes required for the proper function of the cilium, basal body, transition zone, and centrosome [16]. Photoreceptors in the outer segment of the retina have specialized cilia, called primary cilia, that act as sensors for signals from the extracellular environment, such as light, chemical, or mechanical stimuli [17]. The disruption of the BBSome is a well-established cause of underlying syndromic ciliopathies, such as BBS [3,8]. Among these proteins, BBS9 acts as the core element of the BBSome by recruiting the other members of the complex [6,7].

Biallelic variants in the *BBS9* gene cause a multisystemic disorder named BBS9 (MIM: 615986). Its cardinal features are retinal cone-rod dystrophy, obesity, postaxial polydactyly, cognitive impairment, hypogonadotropic hypogonadism, genitourinary malformations, renal malformations, and renal parenchymal disease [1,2]. However, the disorder is characterized by substantial clinical variability, even within families [4,18]. Genotype–phenotype correlations have not been outlined due to the small number of reported cases. A significant proportion of affected individuals carrying biallelic truncation variants in *BBS9* shows primary signs of BBS (i.e., retinal dystrophy, bilateral polydactyly, obesity, ID/DD, and genitourinary and hepatic abnormalities). Of note, only a small number of subjects with pathogenic missense variants of the *BBS9* gene has been reported thus far [9,19]. Interestingly, as in the present report, all missense changes have been found to affect the β-propeller domain, and their clinical phenotype appears less severe and mainly characterized by retinitis pigmentosa and polydactyly [9,19,20] (Supplementary Table S2).

We studied the functional impact of the identified *BBS9* variants on patient-derived fibroblasts and showed that they affect the formation and size of the primary cilium, which appears shorter than a healthy donor, suggesting a loss of BBSome assembly. To test this hypothesis, we first examined the stability and degradation of BBS9 mutants in both primary fibroblasts and transiently transfected HEK293T cells. While we did not observe an overt decreased stability associated with the Gly182Val substitution, we documented a defective interaction of the mutant with BBS8/TTC8, in line with the location of the affected residue on the surface of the β-propeller domain directly mediating BBS9 binding to BBS8/TTC8. The hypomorphic role of this variant might underlie the relatively mild clinical phenotype observed in the proband. Consistent with this hypothesis, individuals with similar missense changes presented with a milder phenotype characterized by retinopathy and variable polydactyly with or without obesity or multiorgan anomalies [4], suggesting that a narrow spectrum of *BBS9* changes, restricted to the β-propeller domain, may cause a milder phenotype of BBS9. However, a larger cohort of study is required to confirm this hypothesis.

In conclusion, this report highlights the informativeness of WGS in clarifying the clinical diagnosis and genotype of apparent non-conclusive cases, and the challenge of reliably identifying rare intragenic structural variants in the genetic workup of ultra-rare disorders. In this context, the functional validation of these rare variants is necessary to assess their pathogenicity.

## 4. Materials and Methods

### 4.1. Molecular Analyses

#### 4.1.1. WES

Genomic DNAs were isolated from the blood of the subject and his parents using QIAmp DNA blood midi kit (Qiagen, Hilden, Germany) according to manufacturer’s instructions. WES of the trio and data analysis were performed as previously reported [21,22]. DNA was sequenced using Illumina paired-end sequencing technology by means of Sureselect Human All Exon V.7 (Agilent, Santa Clara, CA, USA) kits. WES raw data were processed and analyzed using an in-house implemented pipeline as previously described [23,24], according to the Genome Analysis Toolkit’s (GATK’s) best practices [25]. The UCSC GRCh37/hg19 version of genome assembly was used as a reference for read alignment by means of BWA-MEM [26] tool and the subsequent variant calling with HaplotypeCaller (GATK v3.7) [25]. Variants’ functional annotation was made with the SnpEff v.5.0 [27] and dbNSFP v.4.2 [28] tools. Relevant in silico impact prediction tools, such as Combined Annotation Dependent Depletion (CADD) v.1.6 [29], Mendelian Clinically Applicable Pathogenicity (M-CAP) v.1.0 [30], and InterVar v.2.0.1 [31], were also used. By filtering against our population-matched database (~2500 WES) and public databases (dbSNP150 and gnomAD V.2.0.1), the analysis focused on high-quality rare variants that affect coding sequences and splice-site regions. Sanger sequencing was performed for variant validation and segregation analyses (primers available upon request).

#### 4.1.2. WGS

WGS was performed on a NovaSeq 6000 platform (Illumina, San Diego, CA, USA) with paired end reads of 150 bp, in accordance with the manufacturer’s instruction. Base joint genotype calling and data analyses were performed using Bcl2FASTQ (Illumina). Read mapping to the GRCh38 reference sequence, small variant, and joint genotyping calling were run using Sentieon v.2023-08 (https://www.sentieon.com). SNPs and InDels hard filtering were applied using GATK, Version 3.8.0 (Broad Institute). Detected high-quality variants were first filtered by frequency <= 5% in the in-house WGS population-matched database (>350 WGS). Remaining coding sequence variants were annotated using a custom pipeline based as previously described [23,24]. Briefly, CDS variants were annotated and filtered against public (gnomAD v.2.1.1, https://gnomad.broadinstitute.org) and in-house (>3100 population-matched exomes) databases to retain private and rare (unknown frequency or MAF < 0.1%) variants with any effect on the coding sequence, and within splice site regions. The predicted functional impact of variants was analyzed by Combined Annotation Dependent Depletion (CADD) v.1.6, M-CAP v.1.3, and InterVar v.2.2.2 algorithms [29,31] to obtain clinical interpretation according to ACMG 2015 guidelines. Detected variants in non-coding regions were annotated and prioritized using Genomiser [32] (phenotype data version 2302). Structural variants were detected using DELLY [33] v.1.1.6 and prioritized using AnnotSV [34] v.3.3.2.

### 4.2. Genomic DNA and cDNA Analyses

Genomic DNA was used to validate the missense variant from the proband and parental blood. Skin biopsy of the proband was obtained after informed consent was acquired. Total RNA was extracted from the proband’s fibroblasts and the parents’ blood using the RNeasy Mini Kit (Qiagen, Hilden, Germany) according to the manufacturer’s recommendations. Total RNA concentration and quality were assessed by measuring absorbance at 260 and 280 nm. Reverse transcription was performed using the SuperScript RT Reagent Kit (Invitrogen, ThermoFisher Scientific, Waltham, MA, USA). The region between Exons 9 and 15 of the *BBS9* cDNA was amplified by PCR using specific primers, and the PCR products were separated on a 2% agarose gel. Primer sequences are available upon request.

### 4.3. Structural Analyses

The structures of the BBS subunits 1, 4, 8, 9, and 18 of the human BBSome complex (Protein Data Bank reference, PDB ID: 6XT9, https://www.rcsb.org/structure/6XT9) [35] were used to inspect the functional relevance of the BBS9 Gly182 residue using the UCSF Chimera software version 1.17.3 (https://www.cgl.ucsf.edu/chimera/) [36]. The electrostatic potential used to color the molecular surface of the β-propeller domain of BBS9 was calculated using the PDB2PQR software version 3.6.2 of the APBS biomolecular solvation software suite (https://server.poissonboltzmann.org/) [37].

### 4.4. DNA Cloning and Mutagenesis

The coding sequences of human *BBS9* (NM_001033604.2) and *BBS8/TTC8* (NM_198309.3) were cloned into pCS2+N-3xFlag and pEGFP-C3 vectors, respectively, which are a kind gift from Dr. Nachury, Dr. Sheffield, and Dr. Zhang [5]. The missense mutation was introduced into wild-type *BBS9* by site-directed mutagenesis (QuickChange II Site-Directed Mutagenesis Kit, Agilent Technologies, Santa Clara, CA, USA).

### 4.5. Cell Cultures and In Vitro Studies

Primary skin fibroblasts from the proband and healthy donor, and HEK293T cell lines were cultured in DMEM supplemented with 10% heat-inactivated FBS, 2 mM glutamine, 100 units/mL of penicillin and 100 µg/mL of streptomycin, and maintained at 37 °C in a humidified atmosphere containing 5% CO_2_. Cultured fibroblasts were lysed in RIPA buffer supplemented with phosphatase and protease inhibitors. HEK293T cells were transiently transfected with wild-type and G182A mutant pCS2+N-3xFlag-tagged-BBS9, and pEGFP-C3-tagged-BBS8 expression vectors. Twenty-four hours after transfection, cells were treated with 10 µM MG132 or 50 µg/mL CHX for 8 h or 18 h and then lysed in RIPA buffer supplemented with phosphatase and protease inhibitors. Both lysates were kept on ice (30 min) and then centrifuged at 20,000× *g* (20 min, 4 °C). Supernatants were collected and protein concentration was determined by Quick Start Bradford Dye Reagent (Bio-Rad Laboratories, Hercules, CA, USA) using bovine serum albumin (BSA) as a standard. Whole-cell homogenates were resolved by SDS PAGE and transferred to nitrocellulose membranes (Thermofisher Scientific, Waltham, MA, USA). Blots were blocked for 1 h with 5% non-fat milk powder in phosphate-buffered saline (PBS) containing 0.05% Tween-20 and incubated overnight with rabbit polyclonal anti-BBS9 (1:1000, Invitrogen, ThermoFisher Scientific), mouse monoclonal anti-FLAG (1:1000, Sigma-Aldrich, Steinheim, Germany) or mouse monoclonal anti-Actin (1:1000 Sigma-Aldrich) primary antibodies, and goat anti-rabbit- or anti-mouse-HRP IgG (1:3000, Invitrogen) secondary antibodies. ECL SuperSignal West Femto Maximum Sensitivity Chemiluminescent Substrate detected immunoreactive proteins, according to the manufacturer’s instructions (ThermoFisher Scientific).

### 4.6. Immunoprecipitation Analyses

HEK293T cells were transiently transfected with wild-type and G182A mutant pCS2+N-3xFlag-tagged-BBS9, and pEGFP-C3-tagged-BBS8 expression vectors. Briefly, 48 h post-transfection, the cells were lysed in buffer containing 20 mM Hepes, 50 mM NaCl, 10 mM EDTA pH 8.0, 2 mM EGTA pH 8.0, 0.5% NP-40, and protease inhibitors. Lysates were centrifuged at 15,000× *g* for 30 min at 4 °C, supernatants were collected, and protein concentration was determined as described above. Five hundred micrograms of whole cell extract were incubated with 20 µL of Protein A/G-Agarose beads (Santa Cruz Biotechnology, Dallas, TX, USA) to reduce non-specific background. Cleared and pre-absorbed lysates were incubated with anti-FLAG antibody (0.8 µg/500 µg, Sigma-Aldrich) for 1 h at 4 °C, followed by overnight incubation with 20 µL of Protein A/G-Agarose beads at 4 °C. The beads were collected by centrifugation and washed four times with lysis buffer at 1000× *g* for 5 min. Finally, the immunoprecipitated proteins were eluted with sample buffer by incubation for 3 min at 95 °C. Tagged proteins were detected by western blotting.

### 4.7. Confocal Laser Scanning Microscopy

Primary skin fibroblasts from the proband and healthy donor were cultured in DMEM supplemented with 10% heat-inactivated FBS, 2 mM glutamine, 100 units/mL of penicillin, and 100 µg/mL of streptomycin and maintained at 37 °C in a humidified atmosphere containing 5% CO_2_. Approximately 3 × 10^4^/mL fibroblasts were seeded on glass coverslips and maintained in culture in complete medium for 24 h. After 30 h of starvation, sub-confluent fibroblasts were fixed with 4% paraformaldehyde. Subsequently, cells were stained with rabbit polyclonal anti-ARL13B (1:100, Abcam, Cambridge, UK) and mouse monoclonal anti-pericentrin (1:100, Abcam) antibodies followed by goat anti-rabbit Alexa Fluor 488 and goat anti-mouse Alexa Fluor 594 (1:200, Molecular Probes, Eugene, OR, USA). After staining, coverslips were extensively rinsed and mounted onto microscope slides using Vectashield with DAPI mounting medium (Vector Laboratories, Newark, CA, USA). Analyses were performed in three independent experiments on a Zeiss LSM980 (Zeiss, Oberkochen, Germany) using a 63×/1.4 N.A. oil objective and excitation spectral laser lines at 405, 488, and 594 nm. Five hundred cells were counted for each condition in each experiment, and axoneme length was measured for each cell. Image acquisition and processing were performed as previously reported [38].

## Data Availability

All data generated or analyzed during the current study are included in this published article and its Supplementary Materials. Further inquiries can be directed to the corresponding authors.

## Supplementary Materials

The supporting information can be downloaded at: https://www.mdpi.com/article/10.3390/ijms25158313/s1.
